# Effects of (−)-Epigallocatechin Gallate (EGCG) on Energy Expenditure and Microglia-Mediated Hypothalamic Inflammation in Mice Fed a High-Fat Diet

**DOI:** 10.3390/nu10111681

**Published:** 2018-11-05

**Authors:** Jihong Zhou, Limin Mao, Ping Xu, Yuefei Wang

**Affiliations:** Tea Research Institute, Zhejiang University, Hangzhou 310058, China; zhoujihong@zju.edu.cn (J.Z.); maolm@zjtea.com (L.M.); zdxp@zju.edu.cn (P.X.)

**Keywords:** obesity, brown adipose tissue, thermogenesis, central nervous system, hypothalamus, microglia, neuroinflammation

## Abstract

Obesity is an escalating global epidemic caused by an imbalance between energy intake and expenditure. (−)-Epigallocatechin-3-gallate (EGCG), the major polyphenol in green tea, has been reported to be conducive to preventing obesity and alleviating obesity-related chronic diseases. However, the role of EGCG in energy metabolism disorders and central nervous system dysfunction induced by a high-fat diet (HFD) remains to be elucidated. The aim of this study was to evaluate the effects of EGCG on brown adipose tissue (BAT) thermogenesis and neuroinflammation in HFD-induced obese C57BL/6J mice. Mice were randomly divided into four groups with different diets: normal chow diet (NCD), normal chow diet supplemented with 1% EGCG (NCD + EGCG), high-fat diet (HFD), and high-fat diet supplemented with 1% EGCG (HFD + EGCG). Investigations based on a four-week experiment were carried out including the BAT activity, energy consumption, mRNA expression of major inflammatory cytokines in the hypothalamus, nuclear factor-kappa B (NF-κB) and signal transducer and activator of transcription 3 (STAT3) phosphorylation, and immunofluorescence staining of microglial marker Iba1 in hypothalamic arcuate nucleus (ARC). Experimental results demonstrated that dietary supplementation of EGCG significantly inhibited HFD-induced obesity by enhancing BAT thermogenesis, and attenuated the hypothalamic inflammation and microglia overactivation by regulating the NF-κB and STAT3 signaling pathways.

## 1. Introduction

Obesity is one of the most challenging public health problems all over the world, resulting from an imbalance between energy intake and expenditure, contributing to cardiovascular disease, type 2 diabetes, steatohepatitis, hypertension, hyperlipidemia and other related metabolic syndromes [1,2].

There are two types of classical fat tissues with opposing functions in mammals: white adipose tissue (WAT) and brown adipose tissue (BAT) [3]. WAT is the premier energy depot, whose excessive accumulation is a prominent indicator of obesity [4]. In contrast, BAT is a thermogenic effector with abundant mitochondrial content, which is conferred by uncoupling protein-1 (UCP-1) and dissipates energy through non-shivering thermogenesis to reduce excess fat storage [5]. Inducers of brown fat cell differentiation, such as peroxisome proliferator-activated receptor-γ co-activator-1-α (PGC-1α) and PR domain containing 16 (PRDM16), have been shown to trigger brown adipocyte differentiation, mitochondrial biogenesis and expression of UCP-1 [6]. Recent animal and human studies have revealed that increasing BAT activity could enhance energy expenditure, reduce weight gain, improve insulin sensitivity, and reverse hyperinsulinemia, making it an effective therapeutic approach to combat obesity and its related metabolic disorders [7].

In addition to peripheral tissues, the central nervous system (CNS), particularly the hypothalamus, plays a key role in the homeostatic regulation of energy metabolism. The hypothalamus receives nutritional, hormonal and neural information of the metabolic status from the body, and thereupon coordinates adaptive changes in food intake and energy expenditure [8]. Certain hypothalamic nuclei have been implicated in the regulation of BAT thermogenesis and energy balance, such as hypothalamic arcuate nucleus (ARC), hypothalamic dorsomedial nucleus (DMN) and hypothalamic paraventricular nucleus (PVN) [9]. Among them, hypothalamic ARC, attached to the bottom of the third ventricle, is of crucial importance to integration of peripheral metabolic signals on energy status [10]. Recent research shows that distinct neuronal populations in hypothalamic ARC, such as proopiomelanocortin (POMC) neurons and agouti-related protein (AgRP) neurons, regulate BAT thermogenesis via activation of sympathetic nervous system [11]. These neurons project mainly to second-order neurons in the hypothalamic DMN and PVN, leading to an integrated response on BAT thermogenesis and energy expenditure [12,13,14].

It has also been reported that overnutrition induces metabolic syndrome-related hypothalamic inflammation [15]. In the early stages, hypothalamic inflammation acts as a causal factor to precede the onset of overt obesity, which will be reinforced by the pro-inflammatory cytokines produced in the peripheral tissues with the development of obesity [16]. Microglial cells are the resident macrophages in the CNS [17]. When microglial cells are activated, there is a great release of pro-inflammatory cytokines and mediators, such as tumor necrosis factor α (*TNF-α*), interleukin-6 (*IL-6*), interleukin 1 beta (*IL-1β*), and nitric oxide (*NO*) [18], contributing to neuronal damage and neural network dysfunction in the CNS [19,20]. Recent studies indicate that dietary excess triggers the overactivation and proliferation of microglia in the hypothalamus, leading to hypothalamic inflammation and neuronal injury [21]. Thus, controlling microglia-mediated inflammatory responses could be a practical method of combatting obesity-induced hypothalamic inflammation [22].

EGCG is the major bioactive catechin in green tea with distinct pharmacological properties, including anti-oxidation, anti-inflammatory, anti-cancer, and anti-obesity effects [23,24]. S Klaus et al. showed that EGCG was highly effective and sufficient in combating diet-induced obesity in mice [25]. Several mechanisms have been proposed to explain the anti-obesity effects of green tea, green tea catechins and EGCG, including the modulation of pancreatic lipase, the inhibition of de novo lipogenesis, the oxidation of fatty acid, and the activation of BAT thermogenesis [25,26,27,28,29]. However, these studies mainly focus on the peripheral tissues, mechanistic details of the CNS regulation underlying these effects remain unclear. In this study, we investigated the role of EGCG in the BAT thermogenesis and the hypothalamic inflammation induced by a high-fat diet (HFD). Furthermore, we examined the activation of microglia in hypothalamic ARC, the expression of inflammatory cytokines, and roles of the nuclear factor-κB (NF-κB) and signal transducer and activator of transcription 3 (STAT3) signaling pathways, to explore the underlying mechanisms of the anti-neuroinflammatory effect.

## 2. Materials and Methods

### 2.1. Experimental Materials

EGCG (purity > 95%) was purchased from Huzhou Rongkai Foliage Extract Co., Ltd. (Huzhou, China). Based on previous literatures [25,30,31] and pre-experiment (data not shown), EGCG was given by blending with corresponding diet at *w*/*w* ratio of 1%. Rabbit monoclonal anti-NF-κB, rabbit monoclonal anti-phospho-NF-κB, rabbit monoclonal anti-Stat3, rabbit monoclonal anti-phospho-Stat3 (Cell Signaling Technology, Beverly, MA, USA) and rabbit polyclonal anti–Iba-1 (Wako Pure Chemical Industries, Ltd., Osaka, Japan) were purchased. HRP-labeled goat anti-rabbit IgG was purchased from Servicebio (Wuhan, China). Donkey anti-rabbit Alexa Fluor 488 was purchased from Life Technologies (Carlsbad, CA, USA). Enzyme-linked immunosorbent assay (ELISA) kits for TNF-α, IL-6, and IL-1β were purchased from R&D Systems (Minneapolis, MN, USA).

### 2.2. Animals and Diet

All animal use procedures were reviewed and approved by the Animal Care and Use Committee at Zhejiang University and conformed to the ZJU-201704-1 protocol guidelines, following the National Institutes of Health Guidelines for the Care and Use of Laboratory Animals. Male C57BL/6J mice (4 weeks old) were purchased from Shanghai SLAC Laboratory Animal Co., Ltd. (Shanghai, China). The mice were housed in a temperature-controlled room on a 12-h light/dark cycle and had access to food and water ad libitum. After a 1-week acclimation period, the mice were randomly divided into 4 groups (*n* = 8), fed with a normal chow diet (NCD), a normal chow diet supplemented with 1% EGCG (NCD + EGCG), a 60 kcal% high-fat diet (HFD), and a 60 kcal% high-fat diet supplemented with 1% EGCG (HFD + EGCG) respectively for 4 weeks (dietary composition in Table 1). Food intakes and body weights were measured every week.

### 2.3. Collection of Serum and Tissue Samples

After the experimental period, blood samples were harvested to determine the serum glucose and lipid level. Perirenal WAT (pWAT), epididymal WAT (eWAT), subcutaneous WAT (sWAT) and interscapular BAT (iBAT) samples were weighted after careful dissection. Tissues isolated for gene expression, ELISA and western blot analysis were immediately frozen in liquid nitrogen and stored at −80°C. Tissues isolated for histological analysis were fixed in 4% paraformaldehyde.

### 2.4. Serum Biochemical Analysis

Levels of serum glucose, total triglyceride (TG), cholesterol (TC), density lipoprotein (LDL), and high-density lipoprotein (HDL) were determined using an automatic biochemical analyzer (TBA-40FR, Toshiba Medical, Tokyo, Japan).

### 2.5. Histological Analysis

Adipose tissues were dissected, washed in saline, and immediately fixed with 4% paraformaldehyde for 24 h before being embedded in paraffin. Then, 5-µm sections were prepared and stained with hematoxylin and eosin (H&E) for general morphological observations.

### 2.6. Cold Tolerance Test and Infrared Imaging of Heat Intensity Measurement

Mice after 4 weeks’ treatment were placed in a 4 °C cold chamber for 4h with free access to food and water. Body temperature was measured with a digital thermometer (BAT-12, Physitemp Instruments, Inc., Clifton, NJ, USA). Infrared imaging of heat intensity in mice was recorded with an infrared camera (Therm-App, Opgal Optronic Industries Ltd., Karmiel, Israel).

### 2.7. Quantitative Real-Time Polymerase Chain Reaction (qRT-PCR) Analysis

Total RNA was extracted from the adipose tissues and hypothalamus using the TRIzol™ Reagent (Invitrogen, Carlsbad, CA, USA), and cDNA was subsequently prepared using a high-capacity cDNA reverse transcription kit (Invitrogen, Carlsbad, CA, USA), according to the manufacturer’s instructions. qRT-PCR was conducted on the LightCycler480 real-time system (Roche, Switzerland) using SYBR Green PCR Master Mix (Applied Biosystems, Foster City, CA, USA). Sequences of the primer we used in this study are listed in Table 2. The relative level of gene expression was calculated according to the comparative (2^−ΔΔCT^) method. Relative mRNA levels were normalized to the level of glyceraldehyde 3-phosphate dehydrogenase (GAPDH) mRNA, and compared to control group. Data were generated from three independent experiments in three replicates for each sample.

### 2.8. Western Blot Analysis

Homogenized tissue samples were lysed in RIPA buffer containing 1× Halt™ protease inhibitor cocktail and 1× Halt™ phosphate inhibitor cocktail (Thermo Scientific, Rockford, IL, USA). The lysate was then centrifuged at 12,000× *g* for 15 min at 4 °C to remove the precipitate. Protein concentrations of samples were examined using a BCA protein assay kit (Thermo Scientific, Rockford, IL, USA). Equal amounts of protein were separated by SDS-PAGE and transferred to PVDF membranes (Millipore, Billerica, MA, USA). Membranes were then blocked with 5% non-fat milk in tris buffered saline tween (TBST) for 1 h at room temperature, and incubated with primary antibodies overnight at 4 °C. After primary antibody incubation, the membranes were rinsed three times in TBST followed by incubating with horse radish peroxidase (HRP)-conjugated secondary antibody for 1 h at room temperature. β-actin was used as a loading control.

### 2.9. ELISA Assays

Hypothalamic homogenate was obtained as described above, using the same protein extraction buffer. Quantitative assessment of TNF-α, IL-6, and IL-1β proteins was performed using ELISA kits according to the production specification. Concentrations of inflammatory cytokines were expressed as pg antigen per mg protein.

### 2.10. Immunofluorescence

Mice after 4 weeks’ treatment were anesthetized and perfused through the ascending aorta with phosphate buffer saline (PBS) followed by a cold fixative containing 4% paraformaldehyde (PFA) in PBS. Brains were dissected and immersed in the same fixative at 4 °C overnight, then the fixative was replaced by 30% sucrose in PBS. 30 μm-thick coronal sections were cut on a freezing microtome (CM30503, Leica Microsystems, Germany) and processed as free-floating sections. According to the method described previously [32], the brain sections were blocked with PBS containing 10% normal donkey serum (Jackson ImmunoResearch, West Grove, PA, USA), 1% bovine serum albumen (Sigma Chemical Co., St. Louis, MO, USA), and 0.3% Triton X-100 for 1 h at room temperature and incubated with primary antibody for 72 h at 4 °C. The sections were then incubated with the secondary antibody for 1 h at room temperature. Fluorescent images were captured using a confocal laser-scanning microscope (FV1000, Olympus, Tokyo, Japan) and analyzed using Image J software (NIH, Bethesda, MD, USA). 

### 2.11. Statistical Analysis

GraphPad Prism version 7.0 (GraphPad Software Inc., San Diego, CA, USA) and SPSS version 18.0 (IBM Corporation., Armonk, NY, USA) were used for the data display and statistical analysis. Results are presented as mean ± standard error of the mean (SEM). The statistical significance was determined by one-way analysis of variance (ANOVA) followed by Tukey’s post hoc test. Values were considered significantly different at *p* < 0.05, and for multiple comparisons, statistical differences among the groups are indicated with superscript letters.

## 3. Results

### 3.1. EGCG Ameliorates Obesity Induced by HFD

**Figure 1:**
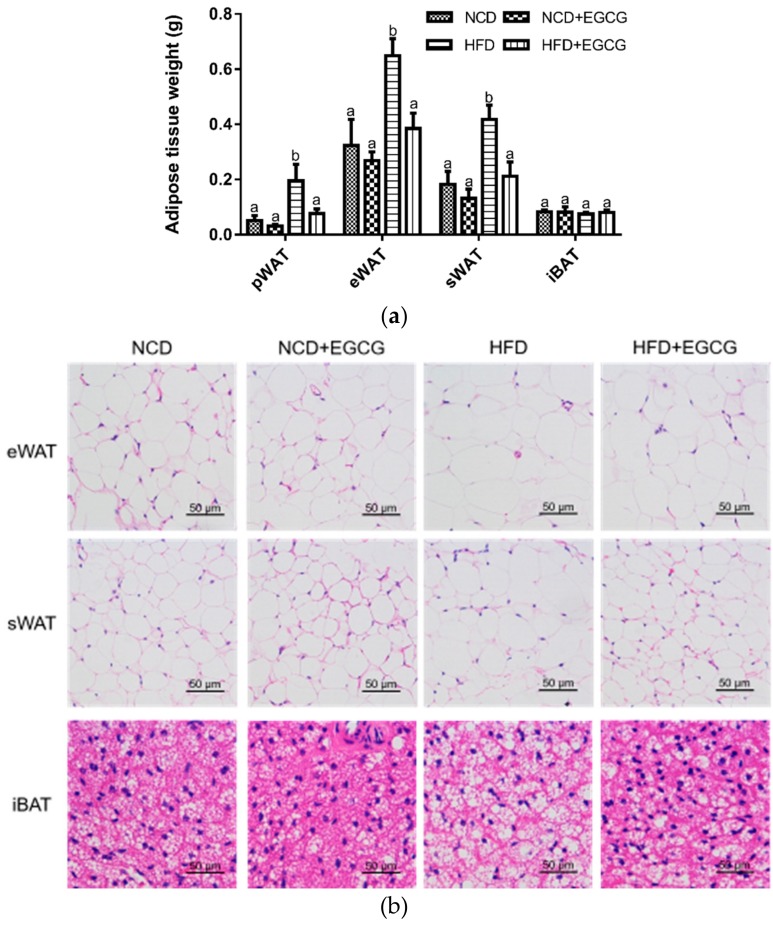
Effect of EGCG on adipose tissue. (**a**) Weight of pWAT, eWAT, sWAT and iBAT; (**b**) Representative hematoxylin and eosin (H&E) staining from eWAT, sWAT and iBAT sections. Data are shown as mean ± SD (*n* = 8). Mean values with different letters (a and b) are significantly different (*p* < 0.05).

Effects of EGCG on the body weight gain, food intake, and serum biochemical parameters are shown in Table 3. After four weeks’ treatment, the HFD induced a marked increase in body weight, while supplementation with EGCG ameliorated this situation without affecting the food and energy intake. Additionally, the HFD + EGCG group showed a sharp decline in blood glucose and TG compared with the HFD group. Supplementation with EGCG also enormously reduced the weights of pWAT, eWAT and sWAT by 60.5%, 40.7%, and 49.3%, respectively, compared with the HFD group (Figure 1a). Meanwhile, histological staining showed an obvious reduction in the size of adipose cells in WAT and a notable suppression in lipid droplets deposition in BAT (Figure 1b). No significant difference was found between the NCD group and the NCD + EGCG group. These results indicate that EGCG treatment reduced the lipid accumulation in adipose tissues and resisted obesity induced by HFD.

### 3.2. EGCG Enhances BAT Thermogenesis

**Figure 2:**
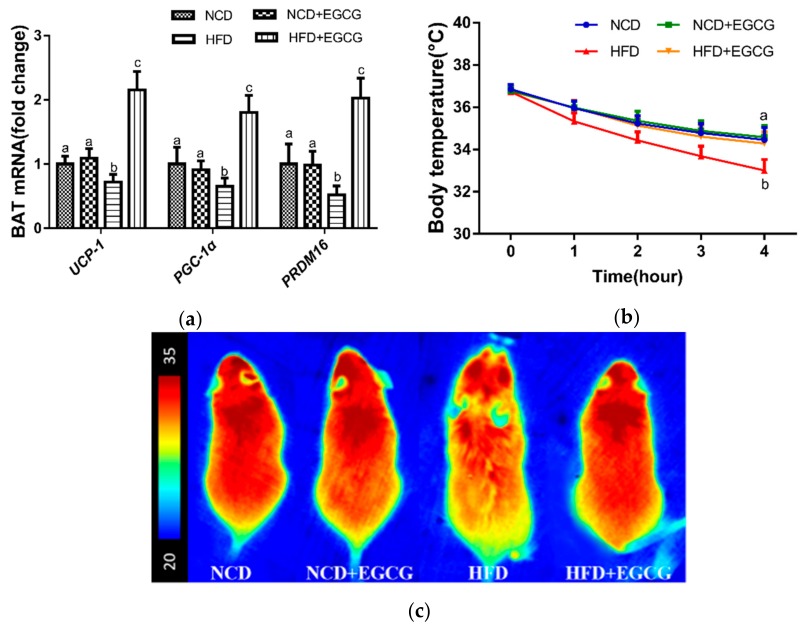
Effect of EGCG on thermogenesis and representative gene expression of BAT. (**a**) mRNA expressions of thermogenic-related gene in BAT; (**b**) Change of body temperature during cold exposure; (**c**) Infrared thermal images of mice after 4-hour cold exposure. Data are the means ± SD of three independent experiments performed in triplicate (*n* = 3). Mean values with different letters (a, b and c) are significantly different (*p* < 0.05).

As mentioned above, EGCG ameliorates HFD-induced obesity without significant changes in food and energy intake, we speculated that EGCG played an important role in energy expenditure through promoting BAT activity. Indeed, mRNA expressions of genes related to thermogenesis and mitochondrial biogenesis in BAT, such as *UCP1*, *PGC-1α*, and *PRDM16* [5,33], were dramatically increased in HFD + EGCG group (Figure 2a). Previous studies have shown that BAT activation induced by excessive energy intake is similar to that induced by cold exposure [6,7]. Therefore, a cold tolerance test was implemented to evaluate adaptive thermogenesis among the four groups of mice [34]. Based on the experiment, it was found that when mice were exposed to a 4 °C environment, body temperatures of the HFD mice dropped significantly compared to those of the NCD mice as time went on, which could be dramatically alleviated by EGCG supplementation (Figure 2b). In accordance with previous studies [35,36], this result suggested that HFD does great damage to the temperature homeostasis regulation, while EGCG helped the body adapt to cold stimulation by producing more heat. Consistently, the infrared thermal imaging results showed that the HFD + EGCG mice could maintain a higher temperature compared to the HFD mice (Figure 2c). No significant difference was found between the NCD group and the NCD + EGCG group. These data indicated that EGCG treatment increased energy expenditure owing to enhanced BAT thermogenesis and resisted obesity induced by HFD.

### 3.3. EGCG Reduces Inflammation in the Hypothalamus

**Figure 3:**
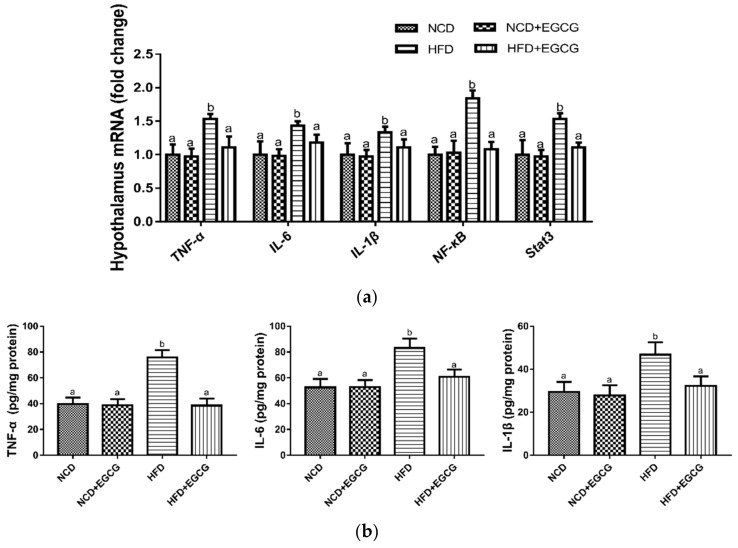

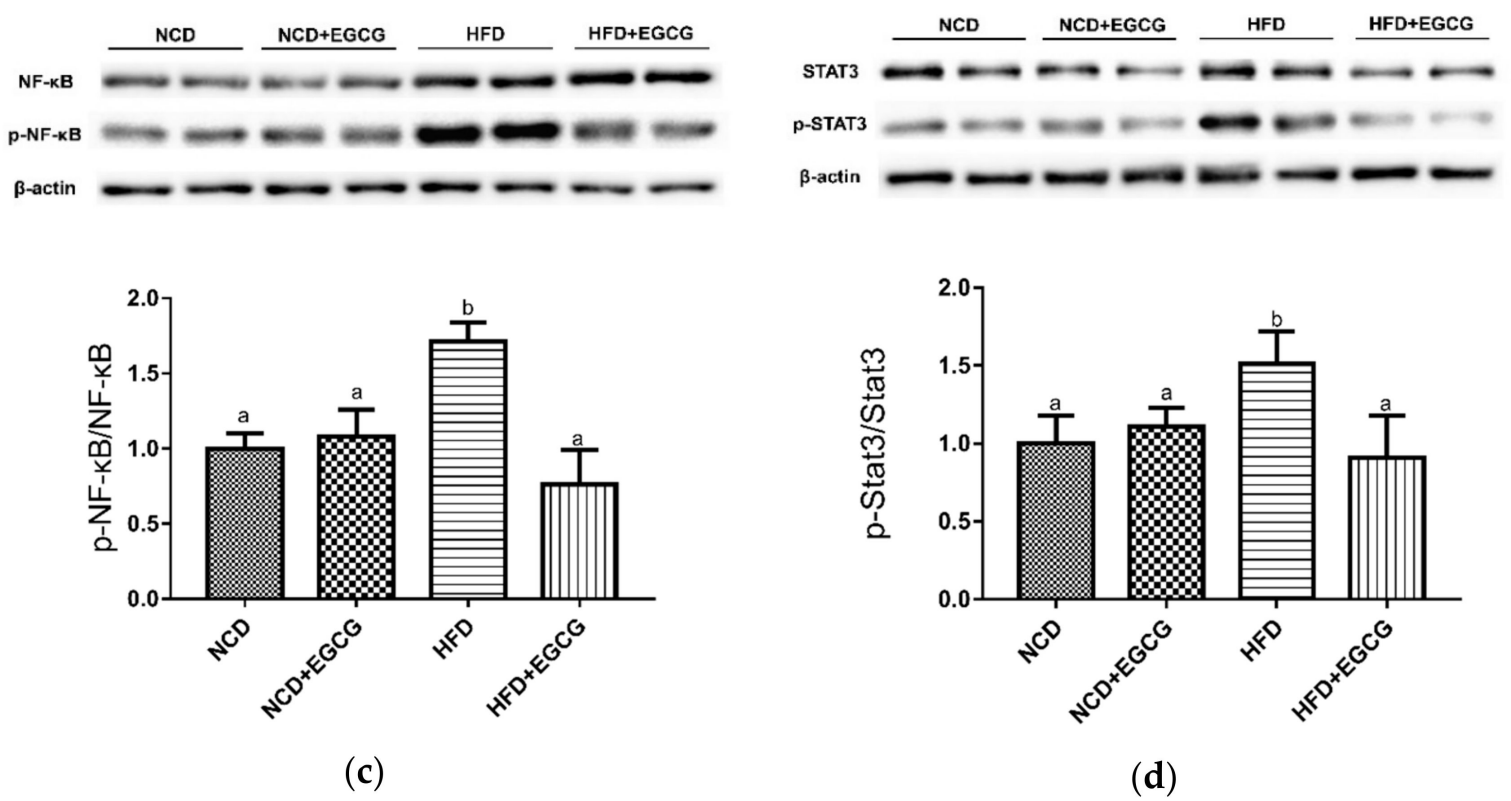
Effect of EGCG on hypothalamic inflammation. (**a**) mRNA expressions of *TNF-α*, *IL-6*, *IL-1β*, *NF-κB* and *Stat3* in the hypothalamus; (**b**) Concentrations of TNF-α, IL-6, IL-1β in the hypothalamus; (**c**,**d**) Immunoblot analysis of NF-κB and Stat3 phosphorylation levels in the hypothalamus. Data are the means ± SD of three independent experiments performed in triplicate (*n* = 3). Mean values with different letters (a and b) are significantly different (*p* < 0.05).

The hypothalamus is the main central structure involved in regulating lipid metabolism and energy balance [37]. Hypothalamic neuroinflammation plays a significant role in HFD-induced obesity [38,39], while the activation of inflammatory cytokines is the key mediator of the inflammatory process and consequent inflammatory impairment [40]. In our study, we found that HFD strongly increased the mRNA and protein expressions of main inflammatory cytokines in the hypothalamus, such as TNF-α, IL-6, and IL-1β, which could be suppressed by supplementation with EGCG (Figure 3a,b). Moreover, western blot analysis of the hypothalamus demonstrated that the expression of p-NF-κB and p-STAT3 is greater in HFD groups compared with NCD groups, while EGCG treatment significantly inhibited the hypothalamic NF-κB and STAT3 phosphorylation (Figure 3c,d). It can be concluded that EGCG inhibited HFD-induced hypothalamus inflammatory responses via suppression of NF-κB and STAT3 signaling pathways.

**Figure 4:**
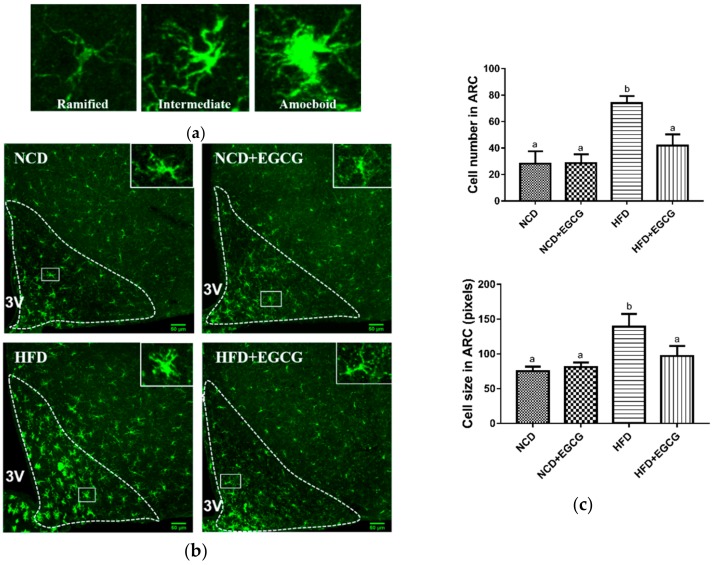
Effect of EGCG on microglia activation in hypothalamic arcuate nucleus (ARC). (**a**) Representative micrographs of three different morphological phenotypes of microglia: ramified, intermediate, and amoeboid in hypothalamus area; (**b**) Representative micrographs of immunofluorescence labeling for Iba1 in hypothalamic ARC (outlined by white dashed lines) and higher magnification insets (outlined by white solid lines) (**c**) Quantification of cell number and cell size of positive Iba-1 cells in hypothalamic ARC. Data are shown as mean ± SD (*n* = 3). Mean values with different letters (a and b) are significantly different (*p* < 0.05).

Activated microglia are known to produce an array of inflammatory cytokines, which may contribute to neuronal death and function imbalance in the CNS [40]. Therefore, we examined the microglial activation level in hypothalamic ARC using immunofluorescence staining of microglial marker Iba1. Microglia can be classified into three phenotypes: ramified, intermediate, and amoeboid, by distinct functional states (Figure 4a). Ramified microglia signify resting, while amoeboid microglia signify activating [41]. Micrographs of immunofluorescence labeling for Iba1 showed that HFD initiated microglial activation in hypothalamic ARC, presenting the typical morphology of activated cells with increased cell number and enlarged cell size. The excessive activation could be inhibited by supplementation with EGCG (Figure 4b,c).

Accordingly, EGCG treatment prevented the expansion and proinflammatory activation of microglia in the ARC and hypothalamic inflammation induced by HFD.

## 4. Discussion

Energy imbalance is one of the main causes of obesity. Reducing energy intake or increasing energy expenditure is widely considered as the effective strategy for weight loss [42]. Recently, by improving the energy expenditure, BAT thermogenesis has gained increasing attention as a potential treatment for obesity and related metabolic disorders [43]. Several studies have reported that the BAT thermogenesis and mitochondrial synthesis could be promoted by polyphenols, such as cyanidin-3-glucoside [35], curcumin [44], rutin [45] and tea polyphenols [46]. Consistent with previous studies [36], this study revealed that EGCG increased energy expenditure and reduced adiposity in HFD-induced obese mice via enhancing the BAT activity, indicating that EGCG could be used to activate BAT and combat obesity in a therapeutic view.

Obesity results from the interplay of many physiological systems; the hypothalamus is considered as a key organ in the CNS to regulate energy balance and homeostatic functions [47]. Substantial evidence indicates that obesity is closely associated with an increase of hypothalamic neuroinflammation, during which microglia are activated to trigger an inflammatory response that affects the functions of key hypothalamic neurons involved in the regulation of energy expenditure [48]. It has been reported that the transcription factor, NF-κB [49], and other signaling pathways, such as mitogen-activated protein kinase (MAPK) [50] and janus kinase/signal transducers and activators of transcription (JAK-STAT) [51] signaling pathways regulate the expression of pro-inflammatory genes in microglial activation. In this study, we demonstrated that EGCG inhibited the hypothalamic NF-κB and STAT3 phosphorylation, and suppressed production and release of the inflammatory cytokines *TNF-α*, *IL-6*, and *IL-1β*, in HFD-induced obese mice. As described above, hypothalamic ARC is a key site of the brain that controls energy balance, associated with specific neurons and neuropeptides released from them. In addition to POMC neurons and AgRP neurons, GABAergic RIP-Cre neurons in hypothalamic ARC were also found to selectively regulate BAT activity and energy expenditure [52]. Immunofluorescence analysis showed activated microglia in hypothalamic ARC of HFD-fed mice, while EGCG reversed this situation. Further investigation is needed into neuron projection development pathways in the hypothalamus and neuropeptide release related to energy metabolism, as well as its precise anti-inflammatory mechanisms.

## 5. Conclusions

**Figure 5:**
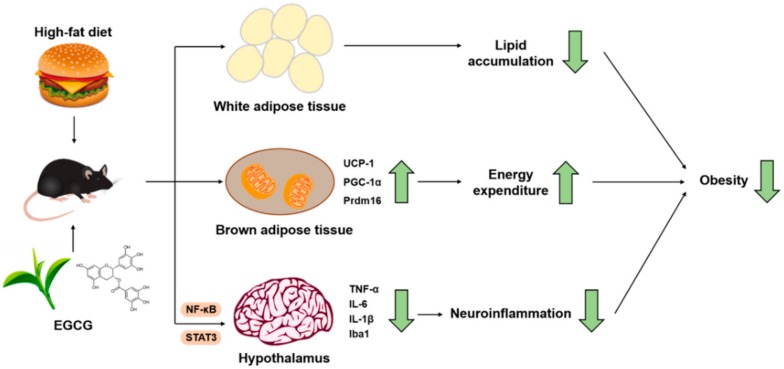
Schematic diagram showing the possible mechanisms of EGCG’s anti-obesity effects.

The present study demonstrated that EGCG could ameliorate obesity by increasing energy expenditure through BAT thermogenesis, as well as attenuating microglia-mediated hypothalamic inflammation. In HFD-induced obese mice, EGCG raised the mRNA expressions of genes related to thermogenesis and mitochondrial biogenesis in BAT; HFD-triggered neuroinflammation in the hypothalamus was also restored by EGCG supplementation by inhibiting the NF-κB and STAT3 pathways, as well as the expression of inflammatory mediators, such as *TNF-α*, *IL-6*, and *IL-1β* (Figure 5). Overall, this study offers additional evidence for the potential application of EGCG in combating obesity and maintaining the energy balance regulation in hypothalamus.

## Figures and Tables

**Table 1:** Compositions of experimental diets.

Ingredient (g)	Group			
	NCD	NCD + EGCG	HFD	HFD + EGCG
Casein	200.00	200.00	200.00	200.00
L-Cystine	3.00	3.00	3.00	3.00
Corn Starch	506.20	506.20	0.00	0.00
Maltodextrin	125.00	119.73	125.00	121.13
Sucrose	68.80	63.52	68.80	64.93
Cellulose	50.00	50.00	50.00	50.00
Soybean Oil	25.00	25.00	25.00	25.00
Lard	20.00	20.00	245.00	245.00
Mineral Mix S10026	10.00	10.00	10.00	10.00
DiCalcium Phosphate	13.00	13.00	13.00	13.00
Calcium Carbonate	5.50	5.50	5.50	5.50
Potassium Citrate, 1 H_2_O	16.50	16.50	16.50	16.50
Vitamin Mix V10001	10.00	10.00	10.00	10.00
Choline Bitartrate	2.00	2.00	2.00	2.00
FD&C Yellow Dye #5	0.04	0.04	0.00	0.00
FD&C Blue Dye #1	0.01	0.01	0.05	0.05
EGCG	0.00	10.55	0.00	7.74
Total	1055.05	1055.05	773.85	773.85
**Total energy**				
Energy, Kcal/g	3.85	3.81	5.24	5.20
Protein, %	20.0	20.0	20.0	20.2
Carbohydrate, %	70.0	69.9	20.0	19.5
Fat, %	10.0	10.1	60.0	60.3

NCD, a normal chow diet; NCD + EGCG, a normal chow diet supplemented with 1% EGCG; HFD, a 60 kcal% high-fat diet; HFD + EGCG, a 60 kcal% high-fat diet supplemented with 1% EGCG; FD&C, the United States Federal Food, Drug, and Cosmetic Act.

**Table 2:** Primer sequences used in quantitative real-time PCR.

Gene	Forward Primer (5′ to 3′)	Reverse Primer (3′ to 5′)
*UCP-1*	CCAAAGTCCGCCTTCAGATC	TCTGTAGGCTGCCCAATGAA
*PGC-1α*	GTCGTGTTCCCGATCACCATAT	CTTTGCGGTATTCATCCCTCTT
*PRDM16*	CACGTCTACGGTGAACGGAA	ATGGGATCCATGAAGAACGGT
*TNF-α*	GACCCTCACACTCAGATCATCTTCT	GCTACGACGTGGGCTACAG
*IL-6*	TCTACTCGGCAAACCTAGTGCGTTA	TTCTGACCACAGTGAGGAATGTCCA
*IL-1β*	TCCAGGATGAGGACATGAGCAC	GAACGTCACACACCAGCAGGTTA
*GAPDH*	GAAGGTCGGTGTGAACGGATTTG	CATGTAGACCATGTAGTTGAGGTCA

*UCP-1*, uncoupling protein-1; *PGC-1α*, peroxisome proliferator-activated receptor-γ co-activator-1-α; *PRDM16*, PR domain containing 16; *TNF-α*, tumor necrosis factor α; *IL-6*, interleukin-6; *IL-1β*, interleukin 1 beta; *GAPDH*, glyceraldehyde 3-phosphate dehydrogenase.

**Table 3:** Effect of (−)-Epigallocatechin-3-gallate (EGCG) on body weight gain, food intake, and serum biochemical parameters.

	NCD	NCD + EGCG	HFD	HFD + EGCG
Initial body weight (g)	21.89 ± 0.87 ^a^	21.92 ± 0.78 ^a^	22.05 ± 1.16 ^a^	22.12 ± 1.02 ^a^
Final body weight (g)	24.82 ± 0.38 ^a^	24.80 ± 1.06 ^a^	27.60 ± 0.83 ^b^	25.09 ± 1.27 ^a^
Body weight gain (g)	2.93 ± 0.61 ^a^	2.88 ± 0.50 ^a^	5.55 ± 0.97 ^b^	2.97 ± 0.47 ^a^
Food intake (g/day)	2.93 ± 0.60 ^a^	3.16 ± 0.40 ^a^	4.61 ± 0.68^b^	4.99 ± 1.05 ^b^
Energy intake (kcal/day)	11.29 ± 2.32 ^a^	12.05 ± 1.52 ^a^	24.14 ± 3.58 ^b^	25.93 ± 5.46 ^b^
Energy efficiency (g gain/kcal consumption)	0.26 ± 0.05 ^a^	0.24 ± 0.04 ^a^	0.23 ± 0.04 ^a^	0.11 ± 0.04 ^b^
Glucose (mmol/L)	4.75 ± 0.47 ^a^	4.29 ± 0.51 ^a^	6.92 ± 0.56 ^b^	5.08 ± 0.30 ^c^
TG (mmol/L)	0.74 ± 0.05 ^a^	0.66 ± 0.09 ^a^	1.01 ± 0.11 ^b^	0.82 ± 0.17 ^a^
TC (mmol/L)	2.69 ± 0.19 ^a^	2.63 ± 0.13 ^a^	2.93 ± 0.14 ^b^	2.86 ± 0.12 ^b^
HDL (mmol/L)	2.81 ± 0.17 ^a^	2.87 ± 0.15 ^a^	2.91 ± 0.13 ^a^	2.86 ± 0.17 ^a^
LDL (mmol/L)	0.43 ± 0.02 ^a^	0.42 ± 0.08 ^a^	0.40 ± 0.04 ^a^	0.41 ± 0.08 ^a^

NCD, a normal chow diet; NCD + EGCG, a normal chow diet supplemented with 1% EGCG; HFD, a 60 kcal% high-fat diet; HFD + EGCG, a 60 kcal% high-fat diet supplemented with 1% EGCG. Data are presented as mean ± SEM (*n* = 8). Significant differences among the groups were assessed by one-way ANOVA with Tukey’s post hoc test. Mean values with different letters (a, b and c) are significantly different (*p* < 0.05).
